# Time to start of cardiopulmonary resuscitation and the effect of target temperature management at 33°C and 36°C

**DOI:** 10.1186/2197-425X-3-S1-A844

**Published:** 2015-10-01

**Authors:** J Dankiewicz, T Cronberg, D Erlinge, H Friberg, C Hassager, J Horn, J Hovdenes, J Kjaergaard, M Kuiper, Y Gasche, T Pellis, P Stammet, M Wanscher, J Wetterslev, MP Wise, A Åneman, N Nielsen

**Affiliations:** Department of Anesthesiology and Intensive Care, Skåne University Hospital, Lund, Sweden; Department of Neurology, Skåne University Hospital, Lund, Sweden; Department of Cardiology, Skåne University Hospital, Lund, Sweden; The Heart Center, Copenhagen University Hospital, Copenhagen, Denmark; Department of Intensive Care, Academic Medical Centre, Amsterdam, Netherlands; Department of Anesthesiology, Rikshospitalet, Oslo University Hospital, Oslo, Norway; Department of Intensive Care, Leeuwarden Hospital, Leeuwarden, Netherlands; Geneva University Hospital, Geneva, Switzerland; Department of Intensive Care, Santa Maria degli Ángeli, Pordenone, Italy; Department of Anesthesiology and Intensive Care, Centre Hospitalier de Luxembourg, Luxembourg, Luxembourg; Copenhagen Trial Unit, Copenhagen, Denmark; University Hospital of Wales, Adult Critical Care, Cardiff, United Kingdom; Department of Intensive Care, Liverpool Hospital, Sydney, Australia; Department of Anesthesiology and Intensive Care, Helsingborg Hospital, Helsingborg, Sweden

## Introduction

The optimal target temperature for comatose patients resuscitated from out of hospital cardiac arrest is unknown. It has been hypothesized that patients with long no-flow times, for example those without bystander CPR would have the most to gain from temperature management at lower temperatures [1]. The generalizability of the TTM-trial [2] has been questioned because of a high fraction of patients receiving bystander cardiopulmonary resuscitation (CPR) (73%) and a median start of basic life support (for patients with bystander CPR) of 1 minute (Interquartile range 1-2 minutes).

## Objectives

The aim of this study was to explore any potential interaction between temperature and no-flow time to investigate whether patients who had longer periods of cerebral ischemia had a better response to the lower target temperature of 33°C in the TTM-trial [2].

## Methods

We analysed data from an international clinical trial randomizing cardiac arrest patients to targeted temperature management at 33°C and 36°C for an interaction between no-flow time and intervention group, with neurological function at 180 days after cardiac arrest as the primary outcome. A cerebral performance category (CPC) score of 1 or 2 was considered a good outcome. The interaction term was included in a multivariate logistic model adjusting for design variables in the TTM-trial.

## Results

**Figure 1:**
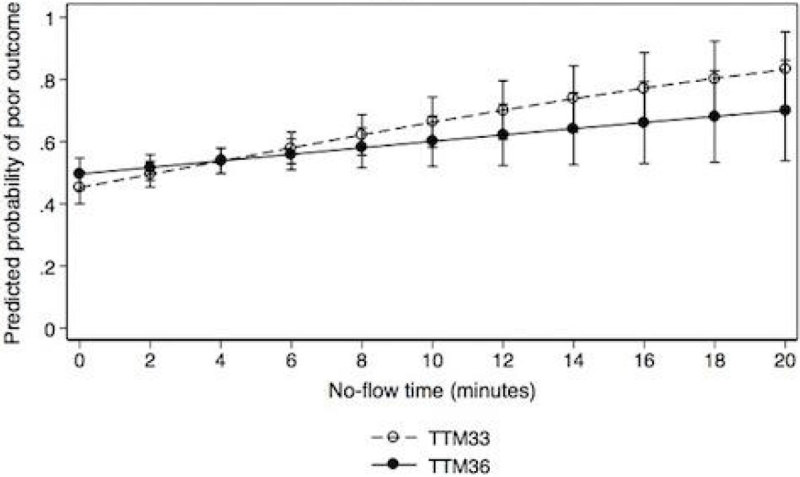
Figure 1

The interaction between no-flow time and temperature group was not significant. Adjusted predictions showed no difference in the probability of a good neurological outcome for any value of no-flow time (Fig 1). In the group of patients with more than eight minutes of no-flow time the difference in the average predicted probability of a poor outcome was -0.018 (95% CI -0.17 - 0.13, p = 0.81) i.e. a non-significant decrease of 1.8% in the probability of a poor neurological outcome for patients treated at 36°C.

## Conclusions

The neutral effect of the two temperature levels was consistent for all no-flow times.

The hypothesis that the efficacy of target temperature at 33°C vs. 36°C is influenced by no-flow time could not be supported.

## Grant Acknowledgment

Supported by independent research grants from the Swedish Heart-Lung Foundation, Arbetsmarknadens Försäkringsaktiebolag Insurance Foundation, Swedish Research Council, Region Skåne (Sweden), Skåne University Hospital, TrygFonden (Denmark), and European Clinical Research Infrastructures Network.
